# Nursing roles in disaster zones: Experiences and lessons from Turkey's 2023 earthquakes

**DOI:** 10.1111/inr.12964

**Published:** 2024-04-11

**Authors:** Ronen Segev, Moriya Suliman, Roee Gorodetzer, Ludmila Zukin, Ahuva Spitz

**Affiliations:** ^1^ Department of Nursing The Stanley Steyer School of Health Professions Faculty of Medical and Health Sciences Tel‐Aviv University Tel‐Aviv Israel; ^2^ Intensive Care Unit Sheba Medical Center Ramat‐Gan Israel; ^3^ Intensive Care Unit Shaare Zedek Medical Center Jerusalem Israel; ^4^ Head Nurse, Israel Defense Forces Medical Corps Tel HaShomer Israel; ^5^ Department of Nursing Jerusalem College of Technology Jerusalem Israel

**Keywords:** Collaboration, critical care nursing, disaster, emergency nursing, fieldwork, humanitarian aid, multicultural team

## Abstract

**Background:**

Disasters affect human health and well‐being globally. Nursing plays a vital role in disaster preparedness and response, ensuring efficient early care coordination and delivering effective field treatment.

**Aim:**

This study investigates the challenges an Israeli humanitarian delegation encountered during their response to major earthquakes in Turkey in 2023. It explicitly focuses on difficulties in preparation, operations, and collaboration with local teams. The study further analyzes the findings and extracts valuable lessons from the mission.

**Methods:**

Using a qualitative descriptive design, 22 out of 32 nurses involved in delegation participated in three focus group discussions within two months of returning to Israel. The discussions were recorded, transcribed verbatim, and analyzed thematically. The study followed the COREQ guidelines, ensuring comprehensive reporting and methodological rigor in qualitative research.

**Findings:**

The study's main findings spanned predeparture preparation, mission challenges in the disaster zone, and postmission lessons, each highlighted by subthemes and participant quotations. A strong sense of mission was evident among the participants, along with frustration at inefficient time management prior to deployment. Many participants noted additional challenges, related to the difficulty of working in multiple languages and across cultures, and the opportunities for resolution. Finally, participants called for better psychological support following the mission.

**Conclusion:**

Nurses in disaster zones offer valuable insights to enhance preparation, cross‐cultural communication, and postmission implementation.

**Nursing and health policy implications:**

Nurse managers and healthcare policymakers can utilize this study's findings to develop future nursing training programs in disaster‐related skills. Additionally, it can help foster collaboration among international healthcare teams.

## INTRODUCTION

Natural and man‐made disasters have multiplied significantly worldwide in recent years. Swift responses are crucial for saving lives (Li et al., 2023). The two most powerful earthquakes in modern Turkish history struck Turkey's Kahramanmaraş region in February 2023, causing approximately 57,000 fatalities (Hussain et al., 2023).

Throughout the healthcare system, nurses play a crucial role, particularly in emergency field hospitals (Pourvakhshoori et al., 2017; Segev, 2022). Their clinical and managerial skills are essential, allowing them to provide care before, during, and after crises, resulting in a 50%−70% reduction in injury deaths (Firouzkouhi et al., 2021). Despite recognition for their rapid responses, excellent communication skills, and innovative problem‐solving in disaster zones (Xue et al., 2020), persistent gaps in nursing preparedness training (Labrague et al., 2018; Scrymgeour et al., 2020; Taskiran & Baykal, 2019) can be remedied (Al Harthi et al., 2020) improving the development of nursing expertise in handling different disasters (Labrague & Hammad, 2023; Su et al., 2022).

The Israel Defense Forces Medical Corps (IDF‐MC) has gained significant expertise in deploying humanitarian delegations and setting up field hospitals in disaster‐stricken areas since the 1953 Greece earthquake (Alpert et al., 2018). Between 2010 and 2016, IDF‐MC successfully operated six humanitarian hospitals worldwide (Glick et al., 2016).

On February 8th, 2023, the IDF‐MC delegation responded to the Turkish disaster, deploying a team of 58 physicians, 32 nurses, five paramedics, 15 laboratory and imaging technicians, and 23 administrative staff (The IDF Medicine Corps, 2023) to set up operations in a nearby hospital building, closely collaborating with local medical staff. Successful operation of a foreign field hospital requires efficient logistical planning, appropriate equipment usage, adaptation to a foreign environment, and overcoming cultural and language barriers (Alpert et al., 2018; Bar‐On et al., 2013). While some foreign medical delegations establish independent field hospitals without local medical equipment and infrastructure (Naor & Bernardes, 2016), this study's delegation integrated seamlessly into an existing medical facility.

## STUDY AIM

This study's primary objective is to provide a comprehensive description and analysis of the real‐time challenges faced and insights gained by the nurses in the IDF‐MC delegation. Hopefully, the valuable lessons derived from their experiences can be applied in similar situations internationally.

## METHODS

### Research design

This study utilized a qualitative descriptive approach to elicit and investigate participants’ perspective in disaster responses (Bradshaw et al., 2017). Employing focus groups, a well‐established method known for effectively eliciting detailed descriptions of experiences through interactive group discussions (Sim & Waterfield, 2019), it encouraged participants to generate ideas and share sensitive information. Both are proven benefits of focus groups over individual interviews (Kruger et al., 2019).

Three 60‐ to 90‐minute focus groups were conducted via Zoom between March and May 2023. An interview guide included questions about prior preparation, individual roles in the delegation, challenges encountered, and problem‐solving approaches. To minimize potential biases, we engaged two authors with extensive experience in qualitative interviewing but not affiliated with the delegation but possessed. One commenced each session by introducing the research team and clarifying the study's objectives, while the other guided the conversation. All interactions were documented through audio and video recordings and later transcribed verbatim. The authors adhered to the COREQ 32 reporting checklist throughout (Tong et al., 2007).

### Participants and settings

Using a list of all 32 delegation nurses, we contacted them by telephone. Our sample included only willing nurses actively involved in delegation; 22 agreed to participate in one of three possible Zoom focus groups on specific dates. The smaller‐medium group size (7, 4, and 11, respectively) facilitated meaningful interactions and productive discussions, while the Zoom format allowed participation from various locations nationwide. Only participants and the researchers were present during the interviews. To encourage an open exchange of ideas, the second focus group included nurses with high military ranks, mitigating the potential influence of hierarchical figures. Ten men and 12 women, all with backgrounds in critical care or midwifery, participated (see Table 1).

**TABLE 1:** Focus groups (FG) participants' characteristics.

Variable	FG 1 (*n* = 7) *n* (%)	FG 2 (*n* = 4) *n* (%)	FG 3 (*n* = 11) *n* (%)
Gender			
Male	4 (57.2%)	1 (25%)	5 (45.5%)
Female	3 (42.8%)	3 (75)	6 (55.5%)
Gender (M:F ratio)	4:3	1:3	5:6
Average age (mean)	41 years	43 years	39 years
Academic degree			
B.A.	4 (57.2%)	1 (25%)	8 (73%)
M.A.	3 (42.8%)	3 (75%)	3 (27%)
Nursing seniority (mean)	14.2 years	20.8 years	12.5 years
Participation in previous delegations			
1 delegation	5 (71.4%)	2 (50%)	9(82%)
2 delegations	–	1 (25%)	2(18%)
3+ delegations	2 (28.6%)	1 (25%)	–
Military service status			
Reserve	5 (71.4%)	–	8 (73%)
Regular	2 (28.6%)	4 (100%)	3 (27%)
Clinical setting			
ICU^a^ nurses	3 (42.8%)	3 (75%)	6 (54.5%)
OR^b^ nurses	1 (14.3%)	1 (25%)	–
ED^c^ nurses	2 (28.6%)	–	4 (36.4%)
Midwifery	1 (14.3%)	–	——
Nephrology	–	–	1 (9.1%)

^a^ Intensive care unit.

^b^ Operating room.

^c^ Emergency department.

### Data analysis

Translating qualitative research data into English risks losing the textual meaning. To mitigate this, we maintained the original language for as long as possible (van Nes et al., 2010). Following the Hebrew data analysis, professionals translated the transcripts from Hebrew to English and then back‐translated from English to Hebrew. Subsequently, the research team thoroughly read and re‐read all the transcribed material. Each group was then analyzed individually, with constant comparison analyses to glean further insights (Onwuegbuzie et al., 2009). Each group's findings reinforced known themes rather than discovering new ones.

### Ethical considerations

We sought to mitigate potential ethical concerns when researching care teams engaged in disaster responses and that respect local professional norms and culture (Aacharya et al., 2017; Marutani et al., 2021). All participating nurses affirmed their adherence to local professional norms and cultural practices. Additionally, they were provided with written documentation detailing the study's objectives and were required to sign a consent form, indicating their voluntary participation and agreement to have their responses recorded. Robust anonymization procedures were implemented, giving participants the option to respond to questions at their discretion. The study's content was accessible only to the primary researchers.

Ethical approval for the study was granted by the IDF‐Medicine Corps review board (No. 0902–2023) and the Tel‐Aviv University Ethics Committee (No. 0006518‐2).

### Rigor and integrity

The study's data rigor and integrity were measured applying four criteria: credibility, transferability, dependability, and confirmability, reflecting Krefting's guidelines (1991). To ensure study quality, we implemented several measures including peer examination and member checking of the study design to enhance credibility. Participants provided a detailed description to enhance transferability. We maintained the accuracy of our data collection, analysis, and interpretation methods, while also providing sufficient contextual information to ensure dependability. Lastly, we conducted a thorough rechecking of our findings to ensure confirmability. The primary investigators, both with expertise in qualitative methodology, independently analyzed the data before converging to compare and deliberate on their respective findings. Ultimately, participants were afforded the opportunity to review the findings and validate their precision.

## FINDINGS

The findings provide insights into challenges encountered during the integration process between local and foreign teams, including political tensions, language differences, and cultural and social disparities. Participants expressed an initial feeling of detachment or “otherness,” which gradually evolved into a sense of camaraderie as they engaged in interactions and caregiving experiences with the local team. The study's main findings can be organized thematically on a chronological axis, aligning with the participants’ perception of time (Table 2):

1. Predeparture preparation
2. Challenges when working in a disaster zone
3. Postmission lessons

**TABLE 2:** The study themes and subthemes.

Main theme	Subthemes
Predeparture preparation	A sense of participating in an exceptional mission Time lost Flattening traditional hierarchies
Challenges when working in a disaster zone	Inclement weather Language barriers Difficulties collaborating with local teams
Postmission lessons	Nurse–physician ratio Medical records' language incompatibility Seeking psychological closure

### Theme 1: Predeparture preparation

All participants described the predeployment period as a valuable opportunity to come together, share their enthusiasm for the mission, and engage in thorough preparation. The most prominent positive subthemes were feeling a sense of an exceptional mission and the flattening of traditional hierarchies between delegation members. A negative subtheme was “time lost,” encompassing a sense of the inadequate utilization of the extended predeparture period.

#### Subtheme 1: A sense of participating in an exceptional mission

Delegation nurses felt that any personal uncertainties or concerns were overshadowed by a strong sense of commitment to saving lives at an international level and a feeling of camaraderie. As the following quotes demonstrate, nurses exhibited a strong motivation and willingness to dedicate their time, care, competencies, and skills toward saving the lives of others:

“I immediately jumped at the opportunity; …Curiosity and pride overcame all fears.” (Participant #12)

“I chose to join really from a sense of mission. I think…if you believe in the mission…you just go. No matter what…immediately…I said yes…It was both an honor and a great privilege for me to participate….” (Participant #20)

“I didn't think twice – the last time [I participated in a like delegation] I left a 5‐month‐old baby; and I didn't think this time either. When they asked me, I immediately said yes. First, this comes from a sense of mission; second…it seems clear to me that you are called to the flag.” (Participant #3)

“This [was] not my first delegation…But as soon as there is a task – everything lights up. The strength, the heart, and the energies will all be on the alert… For me there is such a *rush* that you want…to be there already.” (Participant #10)

#### Subtheme 2: Time lost

Although enthusiastic about the mission, participants recognized the critical role of time in saving lives. They shared frustration at the extended time between invitation, team assembly, and actual departure, time many felt could have been utilized more effectively to facilitate team bonding, more thorough briefing, and better preparation:

“We received the alert Monday morning and the final okay around 9–10 pm… We arrived at 8 am…but [departure] was postponed and postponed and postponed and the 24‐hour wait left an impression of disorganization.” (Participant #15)

“We need to use [the departure preparation day] more effectively, even if it only means getting to know who I work with because I did all this myself…If you board the plane and already know who you will be working with, you're at a much better starting point.” (Participant #8)

“There were many hours of waiting…. From the moment we assembled, it took 36 hours until we landed in Turkey.” (Participant #16)

#### Subtheme 3: Flattening traditional hierarchies

One interesting observation of all the interviewees was that, although professional hierarchies are familiar to hospital nurses and physicians, these faded into the background from the start of the mission. Everyone collaborated, irrespective of rank:

“Before we set up the emergency room…I didn't function as an emergency room nurse. I loaded boxes and cleaned containers, assembled air conditioners, built tents. [I was] the person in charge of water and electricity, everyone works with everyone” (Participant #13).

“There's no such thing as ‘Professor’, [or] ‘Lt. Col.’” (Participant #9)

“By the time we arrived at the disaster zone…everyone was equal.” (Participant #14)

The flattened hierarchy enabled closer, more supportive teamwork under uncertain conditions in a foreign work environment:

“Everyone is equal and does everything, right from the beginning. It creates an atmosphere that the whole group is unified; it's an important process.” (Participant #2)

“A mission of destiny…it led me to work with people…better…connect to them, the work really flowed better, and I felt that everyone was pitching in and helping wherever possible….” (Participant #5)

### Theme 2: Challenges when working in a disaster zone

The participants identified various mission challenges, including inclement weather, intercultural challenges like language barriers, and difficulties collaborating with local teams. They had to find effective ways to overcome these challenges in order to provide the necessary assistance.

#### Subtheme 1: Inclement weather

Weather posed significant challenges to the participants upon entering the disaster zone, with the stark differences between their origin country and the destination causing physical shock and discomfort. Participants noted with limited time for physical adaptation to the local environmental conditions, their lack of preparation for these conditions made adjusting even more difficult. The nurses described having to suddenly adjust to new weather and conditions and begin work immediately on arrival:

“The bitter cold was my experience… the first night we slept in tents, and I woke up with ice on my face….” (Participant #9)

“The day we departed; it was super rainy. All the equipment stood outside in the rain until it was put on the trucks…In Turkey, it was also…raining and cold…The tents weren't ready…and there wasn't enough heating equipment.” (Participant #18)

#### Subtheme 2: Language barriers

Beyond the physical challenges, the participants also faced intercultural difficulties, specifically the language barrier with local people speaking only Turkish. However, several Israeli team members spoke Arabic well, enabling them to effectively communicate with the local staff and patients, especially the many Syrian refugees affected by the earthquake. This ability to communicate created a bridge for establishing more intimate and effective care, particularly during the initial interactions in the disaster zone:

“I think that we [nurses] naturally have better communication skills than other professions. Improvisation, body gestures, expressing everything with emotion… [We] noticed it was easier for us to communicate with the Syrian patients in Arabic. We have taken care of Arabic‐speaking patients [professionally] and have some medically oriented Arabic.” (Participant #22)

“Turkish Airlines [English‐speaking, volunteer] staff helped us incredibly. [Not] just with translation; they wanted to help beyond that…reassuring families, reassuring patients, lending a hand, providing water, buying us milk for coffee…It shouldn't be taken for granted that [airline employees] return from a flight and come straight to a hospital to help translate and stay for hours…It really helped. I also think that we learned to communicate with each other.” (Participant #18)

Another way to overcome language issues, the Israeli team was trying to speak in English during shift changes and medical data transfers to enhance collaboration with local teams:

“We decided to speak English as much as possible, especially during patient admission [so] the local senior doctor would understand and write the appropriate orders.” (Participant #6)

The airline workers’ role is especially noteworthy, offering a potential solution in future disasters. Their expertise in navigating different cultures and their wide range of intercultural experiences were valuable assets, facilitating communication and collaboration between the foreign staff and the local teams, and ultimately contributing to a more effective and cohesive working environment.

#### Subtheme 3: Difficulties collaborating with local teams

Operating within existing local healthcare facilities posed significant challenges for the delegation. Differences in working methods and care standards created tensions affecting both the local and Israeli teams. Many described instances when conflicts arose due to these differences, highlighting the importance of effective communication and finding common ground to ensure the smooth functioning of healthcare services in the disaster zone:

“We entered a place, with a certain institutional behavior, [and] way of working. For example, there were differences between us in handling sterile equipment and taking patient histories and doing a physical exam.” (Participant #15)

“The Israeli team would follow a ‘grand rounds’ routine to examine patients. The Turkish team did not participate, [and] made separate rounds…then somehow [the two teams] would try to have a discussion. In the first few days, there was no discussion at all.” (Participant #16)

Initially, there was skepticism and disagreement regarding the Israeli and local teams’ medical approaches. Eventually, the Israeli nurses learned to integrate into the local team and collaborate fully:

“[When we] started working and they [Turkish teams] saw how we insert a catheter into a peripheral vein and dress a wound, they quickly accepted us. The language of professionalism breaks barriers.” (Participant #4)

“I think that after we received the first patient and they saw how we treated him, there was an increase in trust, and you could see it because when there were more difficult cases…they took a step back. The local doctor in charge cried and asked us not to go [back home] because she understood that we were doing good, while having a dialogue with them and good intentions.” (Participant #22)

The Israeli delegation perceived themselves as guests in the disaster zone and recognized the need to take the lead in accommodating to local customs. Despite differences, both teams, as participants related, shared a common goal to provide quality care, which eventually fostered a sense of camaraderie. Additionally, the exchange of medical knowledge provided common ground for collaboration and understanding between the teams.

### Theme 3: Postmission lessons

In contrast to initial challenges, the delegation's departure from the Turkish hospital and the process of transferring information and tools to the local teams proceeded relatively smoothly. Several issues relevant to future delegations emerged from the focus groups, including the optimal ratio of nurses to physicians, medical records’ language compatibility, and seeking psychological closure.

#### Subtheme 1: Nurse–physician ratio

Participants noted that, as a result of high nursing demands, there was an insufficient number of nurses compared to doctors. They expressed the need for a better staffing ratio:

“The numerical ratio between nurses and doctors in the workforce was not so balanced… There were more than enough doctors and too few nurses.” (Participant #16)

“It was already clear before we left Israel that we had a small number of nurses. We knew… [it could] place a significant burden on nurses.” (Participant #18)

#### Subtheme 2: Medical records’ language incompatibility

One of the significant lessons learned from the focus group participants was the need to prepare the medical records language software to accommodate international collaborations. For example, Israeli participants mentioned that their user interface was in Hebrew, making it difficult for local staff to use and severely hampering collaboration:

“The Israeli computerized system…is irrelevant because it's in Hebrew and isn't translated to Turkish. The [patient] documentation…was all in Hebrew, and [the Turkish team] would write notes and try to understand what we wrote.” (Participant #1)

“We worked double time. We recorded in Hebrew and the local team [recorded] in Turkish.” (Participant # 9)

#### Subtheme 3: Seeking psychological closure

Participants observed that they received no emotional preparation prior to departure. While at the disaster zone, ad‐hoc sessions were conducted by a military social worker and the emergency department's head nurse to help them process their experiences, but targeted specific issues only as they arose. The nurses acknowledged that military psychologists checked up on them after their return to Israel. However, despite postmission personal conversations and honorary events, there was a prevailing sense that group closure following their shared experiences was lacking, as was help in processing difficult experiences:

“There was no [psychological] closure, and it was missing. Everyone can talk about it on their own, but no one gathered the group [to talk] … There was a very nice closing event initiated by the medical corps that held an appreciation evening, but there was no room for talking.” (Participant #9)

“[In Turkey], I realized that I didn't feel a lack of psychological support. Upon returning to Israel, I began to feel the absence of such support. This realization came a day or two after my return, as I found myself in a completely different environment. This need … became more significant after the mission. The sudden shift from being in an intense emergency situation, which we often refer to as an inferno, where everything is heightened, to a normal situation was extremely challenging for me to accept. The dissonance between these contrasting experiences was difficult to reconcile.” (Participant #11)

## DISCUSSION

In this study, three main themes emerged, corresponding to the predeparture period, their work in the disaster zone, and postmission lessons. During departure preparations, participants expressed a strong sense of mission in their involvement with the humanitarian aid delegation, corroborating findings from previous international studies on nurses during the predeployment preparation phase (Christensen & Wagner, 2022; Moradi et al., 2020). Participants also expressed frustration with time lost during preparations and recognized the importance of early team building, and the need for more efficient time management. At least one previous study suggested that utilizing structured guidelines and checklists can improve the preparation phase (Erlich et al., 2015). In addition, the flattened hierarchy among delegation members contributed to the team's sense of unity, although we found no mention of this in the existing literature. Together, these findings suggest that the predeparture period would benefit from prioritizing team members’ familiarity with one another in addition to effective predeployment orientation and training (Holmgren et al., 2019).

The second theme that emerged related to challenges faced in the disaster zone. Nurses reflected on the inclement weather, particularly their first cold, rainy night, and the inadequate protection their tents provided. Other challenges included difficulties interacting with the local population, especially with local medical teams. Cultural differences and conflicting perspectives were noted as significant barriers, although there was a concomitant recognition of growing collaboration over time. The recognition of cultural and professional differences among international groups of nurses has been well‐established (Balante et al., 2021). While studies have strongly recommended improving cultural knowledge to enhance collaboration with local medical teams (Chin et al., 2022; Lind et al., 2012), we have not found any studies specifically examining real‐time collaboration between foreign and local teams at a single disaster site.

Consistent with this study's findings, previous literature has recognized extreme weather conditions as a challenge for staff (Hamdanieh et al., 2023). However, unlike in disaster zones where foreign delegations often struggle to find available local buildings or equipment due to extensive infrastructure damage (Naor & Bernardes, 2016), the current delegation had the unique opportunity to enter an existing local health facility. This situation is uncommon and deviates from the typical circumstances encountered in disaster zones.

The third major theme that emerged was the postmission lessons that the nurses shared, including a recommendation to improve the nurse–physician ratio—a finding that contrasts with a previous study calling for more expert physicians in field hospitals (Burnweit & Stylianos, 2011). Additionally, the nurses suggested internationalizing electronic medical records’ language compatibility software, aligning with earlier studies on medical records and charting. These highlighted issues with nursing disaster competency, suggesting a potential relationship between environmental constraints and nursing competency (Yan et al., 2015; Yin et al., 2011).

While previous studies have highlighted the inadequate preparedness of nurses in disaster response and management (Al Harthi et al., 2020; Taskiran & Baykal, 2019), this study provides fresh insights and practical suggestions from nurses on how to address such challenges. The findings suggest that utilizing predeployment time more efficiently, enhancing delegation preparation, fostering team cohesiveness, and addressing concerns related to professional hierarchy are among the strategies that can help overcome these challenges. Additionally, the nurses emphasized the importance of processing the psychological experience after returning from the mission. Studies have highlighted the significance of offering psychological support to teams engaged in disaster relief efforts (Johal & Mounsey, 2017; Sadhaan et al., 2022; Segev, 2022; Xue et al., 2020; Zahos et al., 2022). Despite delegation members receiving some level of psychological support in the disaster zone and upon their return, this study's findings suggest that additional improvements to nurses’ psychological closure are recommended.

## STUDY LIMITATIONS AND FUTURE DIRECTIONS

One potential limitation of the study is its focus solely on nurses’ perspectives. To obtain a more comprehensive understanding of team disaster preparedness, it would be beneficial to include interviews with other professions or logistical disciplines, both foreign and local. This would provide a broader perspective on the topic and enrich the study's findings.

## CONCLUSION

This study highlights the significant role of nursing in emergency disaster relief. Previous studies have shown that nurses, with their diverse experiences and skills, can play a crucial part in designing effective disaster preparedness measures. The findings of this study contribute to the evidence base on emergency response and provide a fresh perspective on the importance of predeployment preparation, cultural sensitivity, and cultural competence during disasters—findings that can be applied to enhance future disaster preparation and interventions.

## IMPLICATIONS FOR NURSING AND HEALTH POLICY

The insights gained from this study can be valuable for nursing managers and educators in improving disaster and emergency nursing competence and enhancing care capabilities in planning and executing future disaster relief programs. Moreover, healthcare stakeholders can benefit from the unique nursing insights provided in this study, particularly as they relate to weather preparedness, effective communication through interoperable software, use of a universal language like English to facilitate collaboration among multinational teams, and the advantage of international emergency‐response collaboration training for local–foreign partnerships.

Furthermore, it is crucial to prioritize psychological preparedness for foreign and local teams prior to disaster zone deployment, at the end of each workday, and in postmission debriefings. These measures are essential for preventing long‐term reactions to unprocessed experiences. We strongly recommend conducting such debriefing sessions with the delegation and local team members through platforms like Zoom, as this can contribute to the closure of the mission experience and foster the development and strengthening of diplomatic relationships.

## AUTHOR CONTRIBUTIONS

Study design: RS, LZ, AS; data collection: RS, MS, RG, AS; data analysis: RS, AS; manuscript writing: RS, MS, AS; critical reading and revisions: RS, MS, RG, LZ, AS; study supervision: RS, AS.

## CONFLICT OF INTEREST STATEMENT

No conflict of interest has been declared by the authors.

## ETHICAL STATEMENT

The study was approved by the IDF‐Medicine Corps review board (No. 0902–2023) and the Tel‐Aviv University Ethics Committee (No. 0006518‐2).
